# Artificial Intelligence-based automated CT brain interpretation to accelerate treatment for acute stroke in rural India: An interrupted time series study

**DOI:** 10.1371/journal.pgph.0003351

**Published:** 2024-07-24

**Authors:** Justy Antony Chiramal, Jacob Johnson, Jemin Webster, D. Rachel Nag, Dennis Robert, Tamaghna Ghosh, Satish Golla, Saniya Pawar, Pranav Krishnan, Paul K. Drain, Stephen J. Mooney

**Affiliations:** 1 Department of Epidemiology, University of Washington School of Public Health, Seattle, Washington, United States of America; 2 Department of Medicine, Baptist Christian Hospital, Mission Chariali, Tezpur, Assam, India; 3 Qure.ai Technologies Pvt. Ltd. Raheja Platinum, Andheri East, Mumbai, India; 4 Department of Global Health, University of Washington School of Public Health, Seattle, Washington, United States of America; Royal Infirmary of Edinburgh, UNITED KINGDOM OF GREAT BRITAIN AND NORTHERN IRELAND

## Abstract

In resource-limited settings, timely treatment of acute stroke is dependent upon accurate diagnosis that draws on non-contrast computed tomography (NCCT) scans of the head. Artificial Intelligence (AI) based devices may be able to assist non-specialist physicians in NCCT interpretation, thereby enabling faster interventions for acute stroke patients in these settings. We evaluated the impact of an AI device by comparing the time to intervention (TTI) from NCCT imaging to significant intervention before (baseline) and after the deployment of AI, in patients diagnosed with stroke (ischemic or hemorrhagic) through a retrospective interrupted time series analysis at a rural hospital managed by non-specialists in India. Significant intervention was defined as thrombolysis or antiplatelet therapy in ischemic strokes, and mannitol for hemorrhagic strokes or mass effect. We also evaluated the diagnostic accuracy of the software using the teleradiologists’ reports as ground truth. Impact analysis in a total of 174 stroke patients (72 in baseline and 102 after deployment) demonstrated a significant reduction of median TTI from 80 minutes (IQR: 56·8–139·5) during baseline to 58·50 (IQR: 30·3–118.2) minutes after AI deployment (Wilcoxon rank sum test—location shift: -21·0, 95% CI: -38·0, -7·0). Diagnostic accuracy evaluation in a total of 864 NCCT scans demonstrated the sensitivity, specificity, positive predictive value (PPV) and negative predictive value (NPV) in detecting intracranial hemorrhage to be 0·89 (95% CI: 0·83–0·93), 0·99 (0·98–1·00), 0·96 (0·91–0·98) and 0·97 (0·96–0·98) respectively, and for infarct these were 0·84 (0·79–0·89), 0·81 (0·77–0·84), 0·58 (0·52–0·63), and 0·94 (0·92–0·96), respectively. AI-based NCCT interpretation supported faster abnormality detection with high accuracy, resulting in persons with acute stroke receiving significantly earlier treatment. Our results suggest that AI-based NCCT interpretation can potentially improve uptake of lifesaving interventions for acute stroke in resource-limited settings.

## 1. Introduction

Stroke is the second leading cause of death and the third leading cause of disability globally [1]. There is a loss of >32,000 neurons per second after stroke, thus time to intervention (TTI) sharply affects the morbidity and mortality in stroke patients [2]. The American Stroke Association guidelines recommend completing a clinical evaluation by neurologist/stroke specialist within 15 minutes, analyzing brain imaging within 45 minutes, and initiating intervention within 60 minutes of patients’ arrival into the Emergency Room (ER) [3]. However, selecting the appropriate intervention requires distinguishing between hemorrhagic and ischemic stroke, since the interventions differ for each. For ischemic stroke, interventions include thrombolysis (if onset ≤4.5 hours), and/or mechanical thrombectomy and antiplatelet agents like Aspirin/ Clopidogrel, after ruling out bleed. For hemorrhagic strokes, interventions like Mannitol or neurosurgical procedures target to reduce intracranial pressure. It is therefore vital that physicians can readily access brain imaging findings, including non-contrast computed tomography (NCCT) scans, which may be the only brain imaging available in many of the Low-and middle-income (LMIC) settings.

Thrombolysis and thrombectomy are not optimally utilized across the world, particularly worse in LMIC settings, that witness increasing incidence and prevalence of stroke, alongside dearth of radiology and neurology expertise [4–7]. Though multiple strategies have been implemented to overcome these barriers, including teleradiology and tele-stroke platforms, recent evidence continues to show incomplete access to and utilization of these platforms and interventions [8, 9].

Artificial Intelligence (AI) based computer-aided diagnostic (CAD) assistance software devices may reduce the TTI in many illnesses like intracranial hemorrhage (ICH), traumatic brain injury, and tuberculosis [10–13]. Our objectives were to evaluate the impact of one such AI that automatically interprets NCCT brain scans in acute stroke. We also evaluated the diagnostic accuracy of the AI in detecting multiple findings in NCCT brain using radiologist report as reference standard (RS).

## 2. Material and methods

### 2.1 Ethics statement

We obtained Institutional Ethics Committee approval from the Emmanuel Health Association, New Delhi (Protocol No:255 [Version 3]) and from the University of Washington, Seattle, United States of America (ID: STUDY00017314).

### 2.2 Study setting

This study was conducted at the Baptist Christian Hospital (BCH), Tezpur, Assam, India, which is a 130 bedded charitable hospital under the Emmanuel Health Association. Anecdotal reports and hospital-based studies indicate a huge burden of young stroke and hemorrhagic type of stroke in Assam. A recent study showed that hemorrhagic strokes accounted for more than 50% of the cases in Assam, compared to only about 20% of the strokes in the rest of India [14]. One of the potential factors for higher incidence of hemorrhagic strokes among the Assamese indigenous population and the tea garden workers could be a high prevalence of hypertension reportedly between 33% to 60.8%, which is the largest single risk factor of stroke [15–18].

The ten-bedded ER at the BCH was managed by the same number of healthcare staff throughout the study period (November 2020-November 2022), with every 8-hour shift covered by one junior medical officer and one consultant physician along with three shift nurses and two support staff. The hospital has a CT scanner; official radiology reports were obtained through a teleradiology service. A regulatory approved AI device (qER from Qure.ai) capable of detecting critical abnormalities on NCCT brain scans was deployed at BCH for the purpose of assisting the non-specialist physicians in interpreting those scans. Though there was a radiologist employed towards the end of the project, he was not involved in emergency or routine reporting of the CT scans as his primary responsibilities were managing other imaging like ultrasonography. Since there were no neurologists at the hospital, the physicians availed of tele-stroke assistance from the Christian Medical College, Ludhiana as a part of physician-based stroke unit model [19].

### 2.3 AI Device description and deployment in study setting

qER (manufacturer: Qure.ai) is an AI-based CAD device that can detect critical findings in NCCT brain scans such as ICH, infarct, mass effect, and midline shift. qER is a hardware agnostic multi-label classifier and has been trained on approximately 300,000 NCCT scans from across the world. In a retrospective validation study, Chilamkurthy etal. observed that qER achieved an AUC of 0·92 (95% CI 0·91–0·93) and of 0·94 (0·92–0·97) for ICH in two different datasets against consensus from a panel of radiologists’ reports as RS. AUCs were 0·93 (0·91–0·94) for midline shift, and 0·86 (0·85–0·87) for mass effect [20]. In a retrospective observational study with data from Swedish Stroke Register, Hillal etal. observed that qER had a sensitivity of 97% in ICH detection against radiologists’ report as RS (n = 1649) [21].

**Fig 1:**
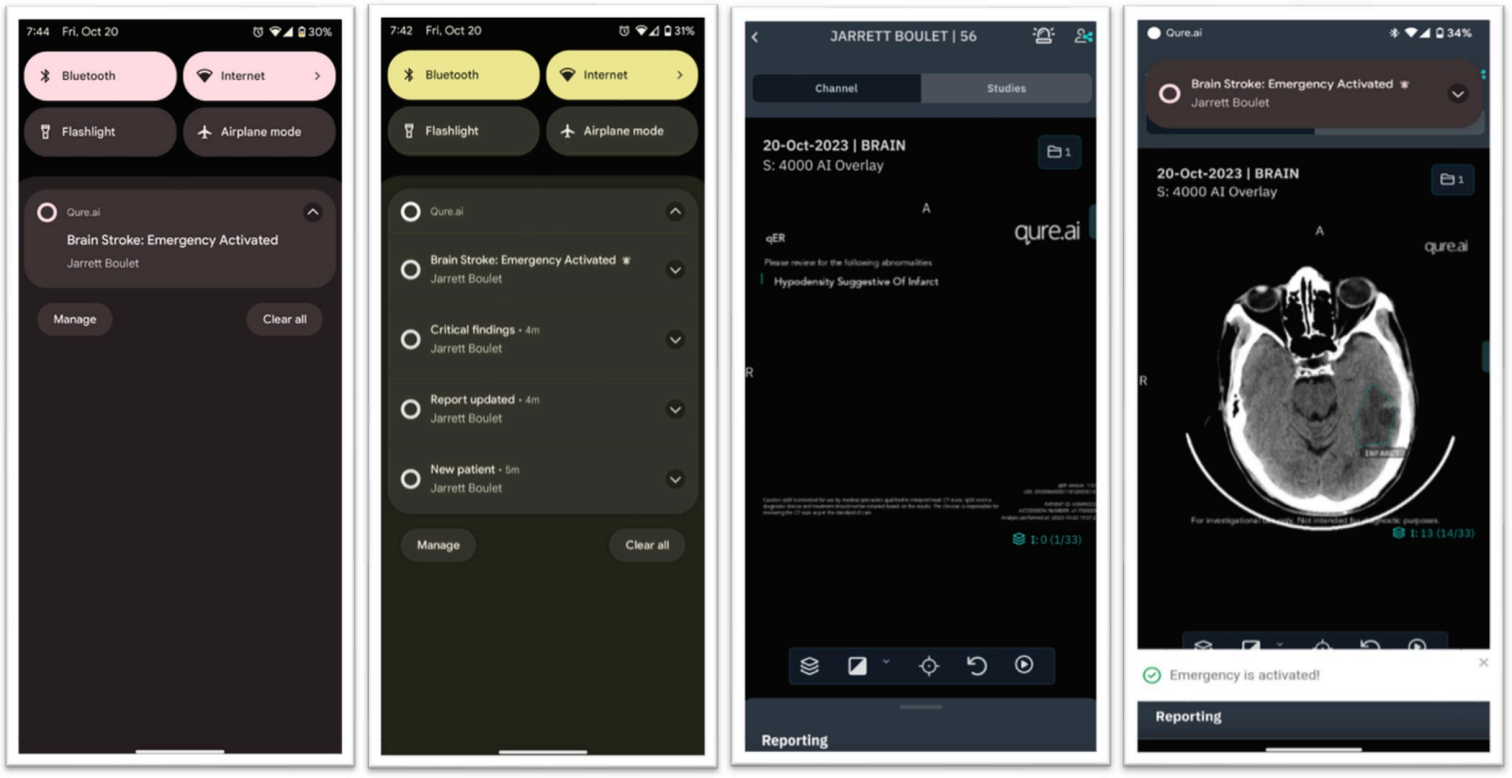
Notification on the phone. A. Top Left: the notification alert on a locked screen; B. Top Right: the notification alert on a screen that is pulled down; C. Bottom Left: the report with findings; D. Bottom Right: key slice with annotations of abnormalities, for e.g. Infarct in the given picture. (Name displayed as patient name is of an imaginary person meant only for demonstrating the user interface).

qER version 2 was deployed at BCH in February 2021, on the computer system connected to the CT machine, by the information technologist at the hospital with guidance from engineers of the AI device manufacturer. During the initial few months (Phase 2) the radiology technician manually uploaded the scans to the cloud. In June 2022, this step was automated (auto-push), instantly uploading to the cloud once the imaging was complete. Once the scan was received, qER processed it and generated a radiology report. A proprietary smartphone application developed by the manufacturer notified and alerted the physicians on their phones within 4–5 minutes of the imaging. This application was HIPAA compliant and served as a Cloud-PACS (Picture Archiving and Communications System) for viewing scans, AI-generated radiology reports, and notifications of annotated abnormalities on the key slices; illustrated in Fig 1. The teleradiologists had no access to the AI report at any point.

### 2.4 Study design and the phases of the study

Our interrupted time series analysis used retrospectively collected clinical data from existing hospital records, radiology reports and AI device outputs to compare the TTI in patients diagnosed with stroke before and after the deployment of the AI. We also evaluated diagnostic accuracy of the AI. We collected data for nine months, which was divided into three phases that spanned three months each.

#### 2.4.1 Pre-AI phase (Phase 1): November 2020 to January 2021 (3 months)

We chose the three months just before the deployment of the software as Phase 1 or the baseline period. There was no in-house radiologist or AI at the hospital during this phase. The standard of care was that the physicians would interpret the NCCT images and confirm the findings with a teleradiologist, whenever it was available. For critically ill patients, if the physicians were not confident of their interpretation from scans, they would contact the on-call teleradiologist to get the report early. For acute ischemic stroke patients who were eligible for thrombolysis, they consulted the tele-stroke team for next steps.

#### 2.4.2 Post-AI phase: Phase 2- January 2022 to March 2022, and phase 3- September 2022 to November 2022

**Fig 2:**
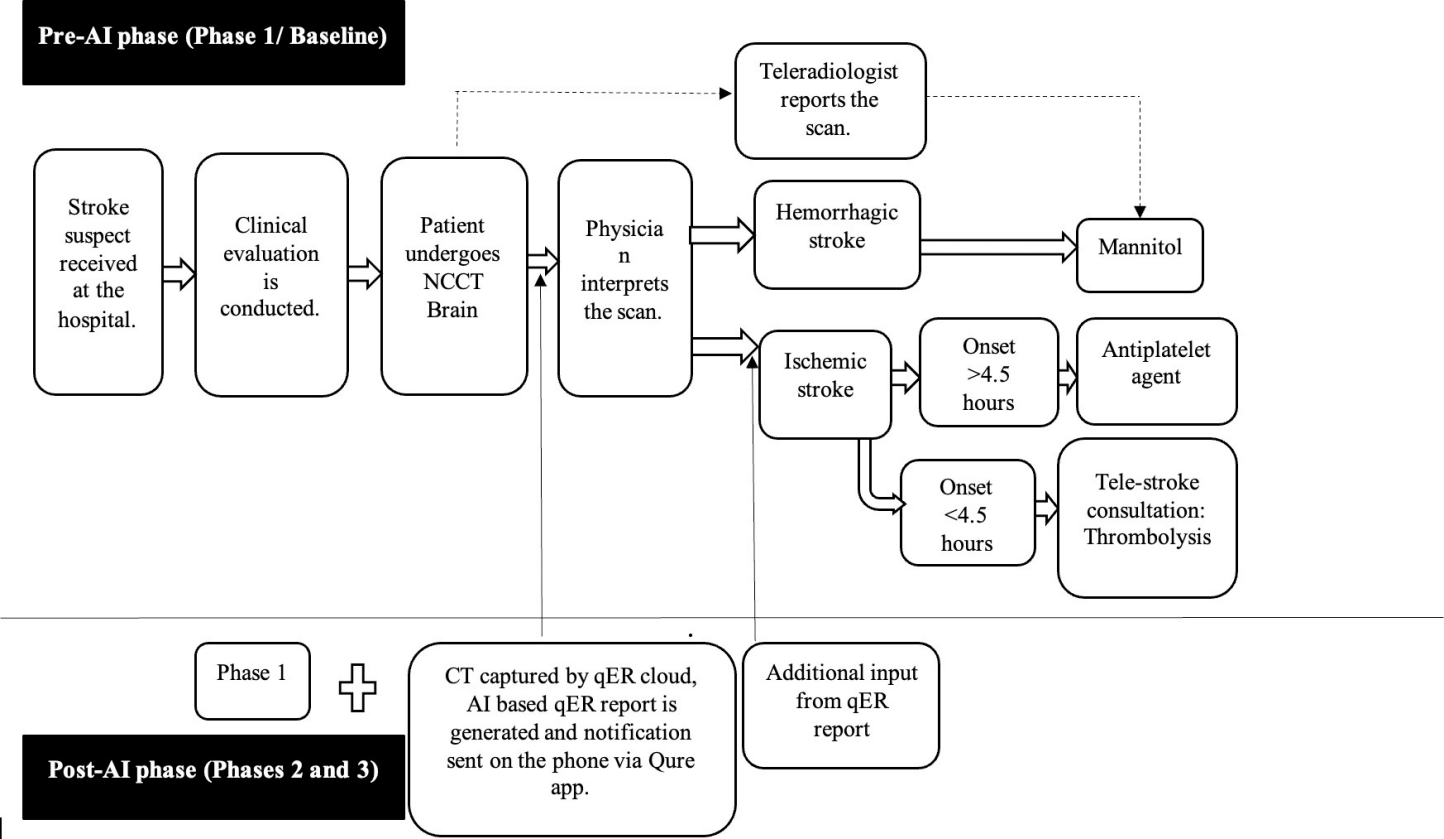
Clinical workflow for suspected stroke patients at BCH, Tezpur in the pre-AI and post-AI phases. Dashed line- represents pathways that were not necessarily part of the clinical decision-making process. Scans were manually uploaded to the cloud in phase 2 and automatically uploaded in phase 3. Description of turnaround times: TTI: from step 3 to step 6 and TTD: from step 3 to step 7.

In post-AI phase (three months each in phases 2 and 3), the clinicians had additional input from the AI report and the notification system, in addition to the standard of care. Auto-push feature was available during phase 3 only. We chose the three-month periods when there was more stable uploading of scans, which was 11 months after baseline for phase 2, and five months after phase 2 for phase 3. This was because of the shift in priorities during pandemic, pandemic related travel restrictions resulting in time lag in tackling technical issues, including the delayed enabling of the automatic upload of CTs on the cloud. Though there was a radiologist posted in BCH during latter half of phase 3, physicians were still dependent on the teleradiologist for the routine and emergency CT reporting. The clinical workflow in both pre-AI and post-AI phases is depicted in Fig 2.

### 2.5 Study population and objectives

All consecutive adults aged ≥18 years who underwent NCCT brain imaging for any indication at BCH during the study period were considered potentially eligible for analysis.

#### 2.5.1 Impact evaluation

The primary objective was an impact evaluation aimed at comparing the time from NCCT imaging to significant intervention (TTI) in patients diagnosed with either hemorrhagic or ischemic stroke before and after deployment of AI. Patients who were diagnosed with acute stroke and had a significant intervention in phases 1 (pre-AI) and 3 (post-AI) were included. We did not include patients from phase 2, because there was only manual uploading of CT scans in that phase, which was dependent on the only radiographer on duty, thus delaying uploading of scans sometimes by hours delaying the AI reports and notifications. Significant intervention was defined as either thrombolysis or antiplatelet agents like Aspirin/Clopidogrel in ischemic stroke or Mannitol for hemorrhagic strokes or for mass effect associated with any kind of stroke. We excluded patients who had diagnoses other than stroke, those with no documented evidence of significant intervention, those with missing date/time for NCCT imaging and/or intervention, those with missing AI reports, and those for whom records indicated significant intervention was before the imaging. As an exploratory analysis, we did a descriptive comparison of time from NCCT imaging to ER disposition (TTD), and scan processing times by AI. TTI and TTD are depicted in Fig 2.

#### 2.5.2 Diagnostic accuracy evaluation

All consecutive patients who had undergone NCCT brain scans for any indication during phases 2 and 3, with available teleradiologist and AI report were included in this evaluation. The primary objective was to estimate sensitivity and specificity of the AI device in detecting four radiological findings in NCCT brain–ICH, infarct, mass effect, and midline shift using teleradiology report as RS. As secondary objectives, we estimated positive predictive value (PPV), negative predictive value (NPV), and kappa statistics for agreement between AI and teleradiologist. Definitions of the outcome measures and radiological findings used for both the impact and diagnostic accuracy evaluations are described in Table 1.

**Table 1:** Definitions of the terms and measures for each objective.

No:	Term	Definition
**IMPACT EVALUATION**		
1	TTI: NCCT imaging to significant intervention	The time from obtaining the NCCT brain scan to the time when the significant intervention, that was dependent on the CT report would be given, either thrombolysis or antiplatelet agents in ischemic stroke and Mannitol for hemorrhagic stroke or mass effect in any type of stroke.
2	TTD: NCCT imaging to disposition from ER	The time from obtaining the NCCT brain scan to the time when the patient was shifted out of the ER which could be either to ICU or ward in the hospital or when referred to another hospital or when the family took the patient home.
**DIAGNOSTIC ACCURACY EVALUATION**		
1	Infarct	Subacute or acute or chronic infarcts or lacunar infarcts or encephalomalacia without a clear non-infarct cause. We excluded scans with only chronic small vessel ischemic changes or periventricular hypoattenuation or chronic microangiopathic changes from labelling as infarct as these entities are radiologically and clinically distinct from infarct.
2	ICH	If there was explicit mention of ICH.
3	Mass effect	If there was explicit mention of mass effect or effacement of anatomical structures mentioned in the report, then we coded the scan as positive for mass effect.
4	Midline shift	If there was explicit mention of midline shift, then the scan was considered to be positive for midline shift.

### 2.6 Data collection

The data were collected by a staff nurse (DRN) at the hospital, on Microsoft Excel spreadsheets, and included demographic details, clinical and radiology reports and time stamps, without patient identifiers. The data collection for this study was initiated on January 5^th^, 2023, from the electronic health records, PACS archive, and the physical records of the patients. Only authors from BCH, Tezpur- JJ, JW and DRN, had access to the database with the patient identifiers. The rest of the authors only had access to the excel sheets with the numeric hospital number, which could not be used to identify patients after data collection since the access to the electronic health record system or PACS or physical records is restricted to the hospital staff alone.

### 2.7 Data analysis

#### 2.7.1 Impact evaluation

JAC and DR have formal medical training and independently identified cases of acute stroke based on clinical features including time of onset, comorbidities, radiology report, final diagnosis, and interventions by manually reviewing the retrospectively collected data from phases 1 and 3. Discrepancies were resolved by consensus meetings and in consultation with the physician from BCH (JJ). Descriptive statistics and comparisons of the distribution of patients in 30-minute time intervals are reported for TTI and TTD. We used the Wilcoxon Rank Sum test to compare TTI.

TTI was investigated additionally using multivariable regression models by fitting TTI as the dependent variable and adding age, gender, type of significant intervention and phase of the study (pre- or post-AI). We used quantile regression models by fitting 50^th^ (median) quantile of TTI [22]. Additionally, 25^th^ and 75^th^ quantile regression models were fitted as part of exploratory analyses, and standard errors were estimated using bootstrapping [23].

#### 2.7.2 Diagnostic accuracy evaluation

We used the teleradiologist report as RS for four target findings (ICH, infarct, mass effect and midline shift). JAC and DR reviewed the radiology reports manually and ascertained the presence or absence of target findings for each NCCT brain scan in phases 2 and 3. In cases of discrepancies, a radiologist (TG) with 5 years of experience was consulted for review of reports and original scans, and his decision was considered the ground truth. Point estimates of sensitivity, specificity, PPV and NPV and 95% exact confidence intervals (CI) for each target finding are reported. Agreement between teleradiologist and AI were estimated by percentage agreement, Cohen’s Kappa and Prevalence-adjusted and Bias-adjusted Kappa (PABAK) along with corresponding 95% CI [24].

Statistical analyses for both impact and diagnostic accuracy were independently performed by JAC and DR, and results were finalized after multiple consensus meetings between them. The study is reported following the STARD guidelines, the checklist is available in the supplementary material (S1 Appendix) [25, 26]. All analyses were done using R version 4.2.1 [27].

Though this was human subject research the need for informed consent was waived since the study was done using retrospectively collected data that was anonymized. All the patient identifiers including contact details were removed in the data spreadsheets, except for the hospital numbers that were required to track the radiology and AI reports.

## 3. Results

Over a nine-month period, 1313 adults underwent NCCT imaging during the pre-AI (n = 323) and post-AI study phases (330 and 660 patients in phases 2 and 3 respectively). There were 174 acute stroke patients for impact evaluation and 851 patients with scans for diagnostic accuracy evaluation. The baseline characteristics of the study population for both impact and diagnostic accuracy evaluation are detailed in Table 2.

**Table 2:** Baseline characteristics of the study population.

	Impact evaluation		Diagnostic accuracy evaluation
Characteristic	Pre-AI (Phase 1) (n = 72)	Post-AI (Phase 3) (n = 102)	Phase 2 and Phase 3 (n = 851)*
**Age**			
Mean (SD)	60·4 (13·2)	62·1 (13·9)	61·4 (13·6)
18–30 years (%)	0	1 (1)	109 (12·8)
31–40 years (%)	4 (5·6)	4 (3·9)	104 (12·2)
41–50 years (%)	10 (13·9)	14 (13·7)	153 (18·0)
51–60 years (%)	20 (27·8)	23 (22·5)	178 (20·9)
61–70 years (%)	21 (29·2)	32 (31·4)	178 (20·9)
>70 years (%)	17 (23·6)	28 (27·5)	142 (16·7)
**Gender**	n (%)	n (%)	n (%)
Female	26 (36·1)	42 (41·2)	365 (42·9)
**Co-morbidities**	n (%)	n (%)	n (%)
Diabetes mellitus	17 (23·6)	18 (17·6)	112 (13·2)
Hypertension	50 (69·4)	78 (76·5)	361 (42·4)
Previous stroke	8 (11·1)	5 (4·9)	36 (4·2)
Previous Heart disease	3 (4·2)	4 (3·9)	12 (1·4)
Smoking	2 (2·8)	5 (4·9)	8 (0·9)
Alcohol consumption	6 (8·3)	11 (10·8)	85 (10·0)
No co-morbidity	13 (18·1)	15 (14·7)	355 (41·7)
**Indication for CT brain**	n (%)	n (%)	n (%)
Acute onset of weakness	29 (40·3)	59 (57·8)	185 (21·7)
Slurred speech	9 (12·5)	6 (5·9)	17 (2)
Trauma	NA	NA	213 (25·0)
Seizure	5 (6·9)	2 (2)	55 (6·5)
Loss of consciousness or altered sensorium	19 (23·6)	26 (25·5)	134 (15·7)
Giddiness	10 (13·9)	7 (6·9)	90 (10·6)
Headache	0	0	68 (8·0)
Other (Vomiting/tremors etc.)	0	0	29 (3·4)
Missing	0	0	73 (8·6)
**Type of Stroke**	n (%)	n (%)	n (%)
Ischemic	39 (54·2)	60 (58·8)	206 (23·8) (Infarct)
Hemorrhagic	33 (45·8)	42 (41·2)	159 (18·4) (ICH)
No stroke	0	0	499 (Others including normal)
**Significant intervention type**	n (%)	n (%)	n (%)
Thrombolysis	1 (1·4)	5 (4·9)	6 (0·7)
Antiplatelet agent	36 (50.0)	55 (53·9)	115 (13·5)
Mannitol	35 (48·6)	42 (41·2)	104 (12·2)
Other	0	0	639 (75·1)
**Status at discharge**	n (%)	n (%)	n (%)
Alive	65 (90·3)	98 (96·1)	654 (76·9)
Died	5 (6·9)	4 (3·9)	19 (2·2)
Referred	2 (2·8)	0	0
Missing	0	0	191 (22·4)

*There were 851 distinct patients with 864 CT scans. 13 patients have had two CT scans, each at different points in time.

### 3.1 Impact evaluation

**Fig 3:**
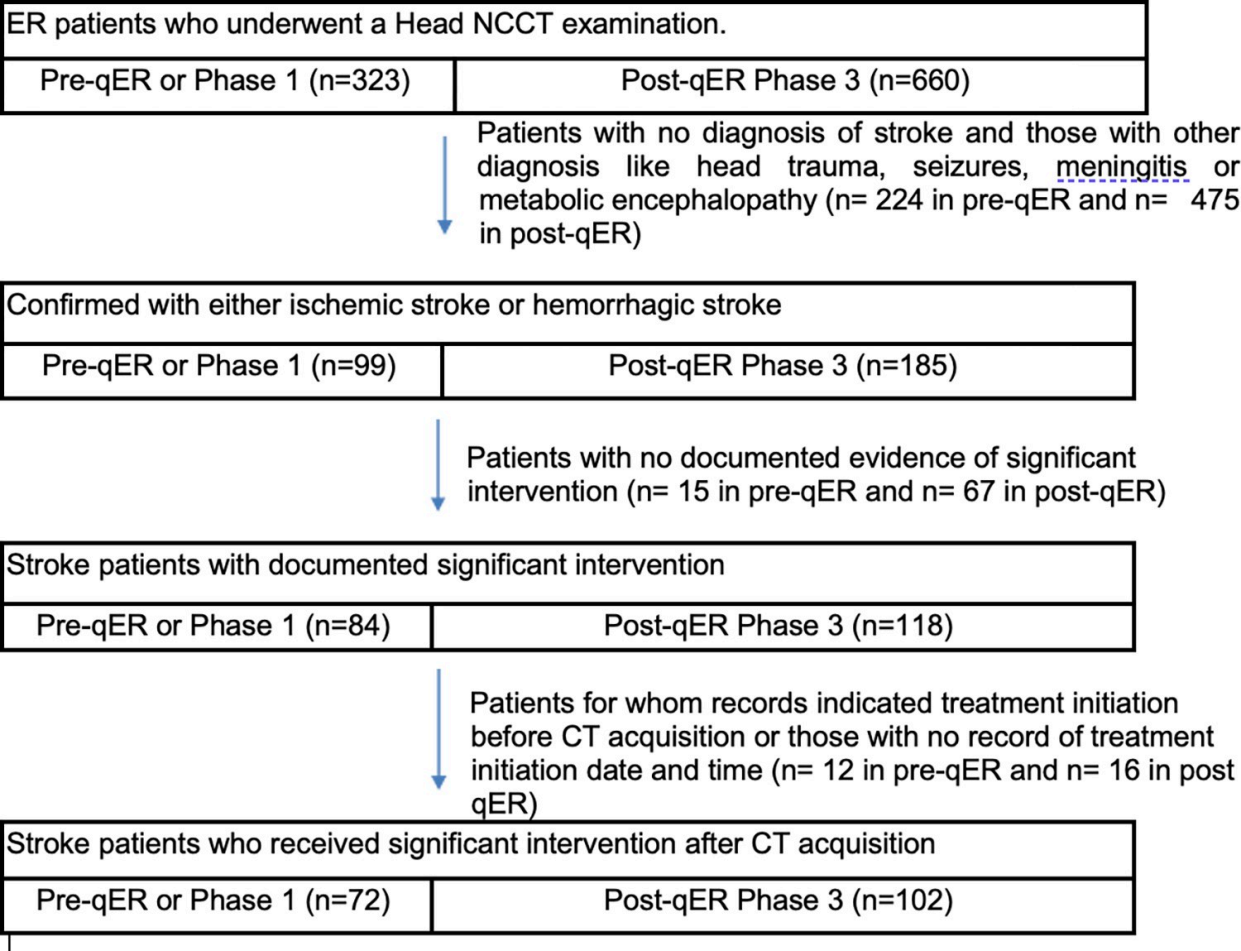
CONSORT diagram for the cohort of acute stroke patients for impact evaluation.

There were a total number of 284 patients in this cohort. After excluding 82 patients with no documented evidence of significant intervention (n = 15 in pre-qER and n = 67 in post-qER) and 28 patients for whom records indicated treatment initiation before CT acquisition or those with no record of treatment initiation date and time (n = 12 in pre-qER and n = 16 in post qER), we had 72 stroke patients in the pre-AI phase and 102 in the post-AI phase (38.7% excluded in total). Fig 3 illustrates the data flow of study participants.

The number of patients diagnosed with ischemic stroke in pre-AI and post-AI phase 3 were 39 (54·2%) and 60 (58·8%) respectively, and the rest of the patients experienced hemorrhagic stroke. 37 (51·4%) patients received either thrombolysis or an antiplatelet therapy as significant intervention in the pre-AI phase compared to 60 (58·8%) in post-AI phase. Thrombolysis was administered to 1 (1·4%) and 5 (4·9%) patients in the pre-AI and the post-AI phases respectively. Overall, 74% had hypertension. 19% of the strokes were young stroke (<50 years age) with one patient in ≤30 years age group.

**Fig 4:**
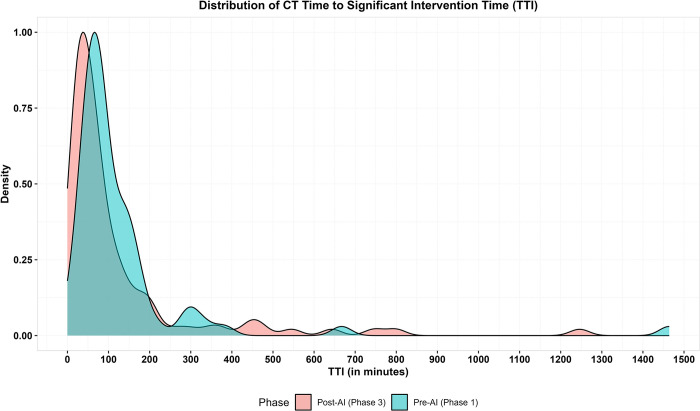
Density plot showing the distribution of time in minutes of TTI, from NCCT imaging to significant intervention for both phases. The peak and the distribution of TTI post-AI is earlier than the peak and distribution of TTI pre-AI.

Descriptive statistics of distribution of patients in each of the 30-minute intervals for the pre-AI and post-AI phases are detailed in Table 3 for the timestamps. Median TTI was found to be 80 minutes (IQR: 56·8–139·5) in pre-AI phase and 58·5 minutes (IQR: 30·3–118·2) and the difference in location shift of TTI was significantly different in post-AI phase based on the Wilcoxon rank sum test (location shift: -21·0, 95% CI: -38·0, -7·0, p: 0·007). Fig 4 shows the distribution of TTI across both phases. The proportion of patients who received significant intervention in less than 30 minutes had increased from 4·2% in pre-AI phase to 25·5% in the post-AI phase (p = 0·0004). Most of the patients in the pre-AI phase received their significant intervention within 30–90 minutes, whereas in the post-AI phase most of them received it within the first 60 minutes after imaging.

**Table 3:** Descriptive summary statistics of impact evaluation.

	Pre-AI (n = 72)	Post-AI (n = 102)
**NCCT imaging to significant intervention in minutes (TTI)**		
Median (IQR)	80 (56·8,139·5)	58·5 (30·3, 118·2)
Mean (SD)	130·4 (188·3)	122·7 (188·1)
Min-max	1–1464	0–1246
0–30 minutes, n (%)	3 (4.2)	26 (25.5)
< 5 minutes, n (%)	1 (1.4)	4 (3.9)
5–10 minutes, n (%)	1 (1.4)	1 (1.0)
10–20 minutes, n (%)	0	5 (4.9)
20–30 minutes, n (%)	1 (1.4)	16 (15.7)
30–60 minutes, n (%)	16 (22·2)	27 (26·5)
60–90 minutes, n (%)	21 (29·2)	17 (16·7)
90–120 minutes, n (%)	9 (12·5)	7 (6·9)
120–150 minutes, n (%)	10 (13·9)	5 (4·9)
150–180 minutes, n (%)	3 (4·2)	3 (2·9)
>180 minutes, n (%)	10 (13·9)	17 (16·7)
**NCCT imaging to disposition from ER in minutes (TTD)**		
Median (IQR)	136 (105·5, 215)	156 (102, 214)
Mean (SD)	100.7 (313·9)	182·5 (240·3)
Min-max	*-1495- 442	*-953- 970
< 30 minutes, n (%)	1 (1·4)	1 (1)
30–60 minutes, n (%)	1 (1·4)	5 (4·9)
60–90 minutes, n (%)	8 (11·1)	10 (9·8)
90–120 minutes, n (%)	13 (18·1)	12 (11·8)
120–150 minutes, n (%)	14 (19·4)	12 (11·8)
150–180 minutes, n (%)	9 (12·5)	15 (14·7)
>180 minutes, n (%)	22 (30·6)	38 (37·3)
Missing, n (%)	4 (5.6)	9 (8.8)

IQR-Interquartile range, SD- Standard deviation

* Some patients had a negative TTD because they were admitted directly from ER to the ward/ICU based on a CT that was done outside BCH. They were included for this objective because there were subsequent scans that were done at BCH, based on which a significant intervention was administered.

In the regression analysis the median TTI was found to be significantly reduced in the post-AI phases (regression coefficient: -21·79, standard error [SE]: 8·57, p = 0·011). Patients who received Mannitol received it faster than patients who received any blood thinners (regression coefficient [beta]: -36·08, SE: 9.90, p = 0·0003). The 25^th^ percentile TTI was also significantly different as per the regression analysis (beta: -26·43, SE: 7·81, p = 0·0008), but the 75^th^ percentile TTI, although reduced in the post-AI phase 3, was not statistically significant (beta: -14·20, SE: 24·06, p = 0·555). More details of the regression analysis are available in the supplementary material (S2 Appendix).

The median TTD showed an increase in post-AI (median: 156, IQR: 102–214) compared to pre-AI phase (median: 135.5 IQR: 104·5–214.5). The median time from imaging to AI report notification was found to be 16 minutes (IQR: 13–19). Within this, the median time for NCCT imaging to acquisition by AI cloud was 12 minutes (IQR: 11–13), and the median time from receiving the NCCT scan by AI cloud to the generation of the AI report was 4 minutes (IQR: 2·25–5).

### 3.2 Diagnostic accuracy evaluation

There were 990 consecutive NCCT brain scans done for any indication, available for analysis from Phase 2 (n = 330) and Phase 3 (n = 660). We excluded two scans with missing AI reports and 124 scans with missing radiology reports (12.7% excluded in total). 124 tele-radiology reports were lost when the hard drive got corrupted during a power surge. Thus, we had 864 eligible NCCT brain scans from 851 distinct patients for this analysis. Thirteen patients each had two NCCT scans taken during multiple visits to BCH. After the independent manual review of the radiology reports of 864 scans by JAC and DR, there were 26 scans that had discrepant RS coding for any of the target findings (ICH, infarct, mass effect and midline shift), and 17 of them were resolved in consensus meetings. TG reviewed the remaining 9 scans along with radiology reports and determined RS for the presence/absence of target findings.

42% of this cohort had hypertension and the most common indication for NCCT imaging was trauma (n = 213, 25%). There were 159 scans with ICH and 206 with infarct. The sensitivity of all findings was ≥0·84 and the specificity were ≥0·93 for all the findings except for infarct. NPV for all findings were ≥ 0·94 and ICH had the highest PPV of 0·96 (95% CI: 0·91–0·98). Cohen’s kappa (k) was 0.90 for ICH suggesting almost perfect agreement between the teleradiologist and AI. There was substantial agreement (0·61< k< 0·8) for mass effect, midline shift, and infarct. All measures of diagnostic accuracy and Kappa statistics with 95% CI for each target finding are reported in Table 4.

**Table 4:** Diagnostic accuracy of the findings for qER from the post-AI phase against radiologist report as reference standard (n = 864).

Finding	No: of abnormal scans	Sensitivity (95% CI)	Specificity (95% CI)	PPV (95% CI)	NPV (95% CI)	% agreement (95% CI)	Cohen’s kappa statistic (95% CI)	PABAK (95% CI)
**ICH**	159	0·89 (0·83, 0·93)	0·99 (0·98,1·00)	0·96 (0·91, 0·98)	0·97 (0·96, 0·99)	0·97 (0·96, 0·98)	0·90 (0·87, 0·94)	0·94 (0·92, 0·96)
**Infarct**	206	0·84 (0·79, 0·89)	0·81 (0·77, 0·84)	0·58 (0·52, 0·63)	0·94 (0·92, 0·96)	0·81 (0·79, 0·84)	0·63 (0·58, 0·69)	0·63 (0·57, 0·68)
**Mass effect**	106	0·86 (0·78, 0·92)	0·93 (0·91, 0·94)	0·62 (0·54, 0·70)	0·98 (0·97, 0·99)	0·92 (0·90, 0·94)	0·68 (0·61, 0·75)	0·84 (0·80, 0·87)
**Midline shift**	80	0·96 (0·89, 0·99)	0·96 (0·95, 0·97)	0·72 (0·62, 0·80)	1·00 (0·99, 1·00)	0·96 (0·95, 0·97)	0·80 (0·74, 0·87)	0·92 (0·89, 0·95)

CI-Confidence Interval, PPV-Positive Predictive Value, NPV- Negative Predictive Value, PABAK- Prevalence-adjusted and bias-adjusted kappa, ICH- Intracranial hemorrhage

## 4. Discussion

Our results support the use of AI for NCCT brain interpretation to treat acute stroke patients more quickly. Our main finding is the significant reduction in median TTI by 21·5 minutes (approximately 30%) for acute stroke patients when using AI. Diagnostic accuracy evaluation demonstrated high performance of AI in terms of high specificity and NPV for all findings. There was a higher burden of hemorrhagic stroke (43%) and hypertension (74%) in this stroke cohort corroborating with existing evidence peculiar to the geographical area of the study site, which is different from other regions of India [14–16].

Our findings of 30% reduction in TTI were significantly different from what Seyam et al. observed when using AI for ICH in a setting where radiologists and neurologists were present. They found a 10% reduction in turnaround time from a baseline of 70 minutes to communicate scans that were positive for ICH [10]. Though there was a radiologist at BCH in latter half of phase 3, since he was not involved with routine and emergency reporting of CT scans, this reduction in time can likely be attributed to AI. We also observed a significant increase in the proportion of patients who got intervention in the first 30 minutes after the imaging in the post-AI phase when compared to the pre-AI phase, 26% vs 4%. We did not observe a reduction in TTD in the post-AI phase which may be dependent on many factors beyond the scope of this study such as the lack of availability of in-patient/ ICU bed or delay in referral. But this serves as a ground reality to show that there was no escalation in ER services/ infrastructure between phases, although they were many months apart.

Our diagnostic accuracy evaluation showed that the sensitivity and specificity of the AI device to detect ICH were 0·89 (95% CI: 0·83–0·93) and 0·99 (95% CI: 0·98–1·00) respectively. Schmitt et al. reported a sensitivity and specificity of 0·91 and 0·89 respectively for a similar AI device in ICH detection [28]. Similarly, Seyam et al. reported sensitivity, specificity, and negative predictive values to be 0·87, 0·94 and 0·98 to detect ICH respectively for another device [10]. The specificity and the NPV of qER to detect ICH were ≥0·97. This is a very crucial finding for deciding on thrombolysis in patients who present within 4·5 hours of onset of acute ischemic stroke, since hyperacute infarct may not be visible on NCCT until many hours later. Diffusion Weighted Imaging sequence of MRI brain is the earliest modality to pick up acute infarcts, which is inaccessible and unaffordable for patients in resource limited settings. Thus, NCCT brain that is negative for ICH in a stroke suspect, could help improve uptake of lifesaving measures like thrombolysis for ischemic strokes in these settings, which is in accordance with the guidelines [3]. There was almost perfect to substantial agreement between AI and radiologist for all of the target findings.

Our results are consistent with the recommendation to use AI in hospitals where there are no in-house radiologists. T.J. Yun and colleagues concluded from a multi-reader diagnostic accuracy project that there was a significant improvement in AUC among physicians with AI assistance, the most noticeable improvement was among non-radiology physicians from 0.92 to 0.95 [29]. qER empowered the physicians as an added boost to their confidence in making early intervention decisions for neurocritical patients. The notification system on their phones alerted them to look at the scans promptly.

The median time from NCCT imaging to generation of AI report was found to be 16 minutes which was unusually high based on experiences from other deployments of the same device. The main limiting factor in this setting was the low internet connectivity, which prolonged the time from imaging to NCCT acquisition by the AI cloud at a median time of 12 minutes. Once the images were acquired, the report was generated within a median time of 4 minutes. A reliable and stable internet connection could enable faster AI report generation.

Our study had many strengths, including analysis from a real-world deployment of a commercially available product in a context where AI can improve time to care. We note below a few limitations. The pre-AI and post-AI phase 3 for impact evaluation were separated by 19 months owing to the COVID-19 pandemic, thus the chance of confounding cannot be ruled out. The effect of the pandemic was also noticeable in the number of patients who underwent CT: phase 3 patients had almost double the number of scans than phases 1 and 2, when hospitals were overwhelmed with COVID patients. Another limitation was that RS for diagnostic accuracy evaluation was based on a single teleradiologist’s report whereas ideal RS should have been collected from a panel of expert radiologists. In the impact evaluation cohort, about 38.7% of potentially eligible patients were excluded mainly due to no documented evidence of administration of a significant intervention. Similarly, about 12.7% of potentially eligible NCCT scans were excluded from the diagnostic accuracy evaluation mainly due to missing radiology reports. These reports were missing from the EMR database, because as per the physicians at BCH the online tele-reporting at night used to come through a different software but was not captured in the EMR. They were saved in a hard drive, which got corrupted during a power surge and thus lost access to this set of reports. Therefore, we cannot exclude the possibility of selection bias affecting our results. Although our study only covered one hospital, we could observe meaningful outcomes from this pilot which is crucial since there have not been any publications that have evaluated the usefulness of this kind of technology in LMICs, where stroke care has been dismal [8, 9].

## 5. Conclusion

Our work demonstrated that using AI that has high accuracy for NCCT interpretation had positive impact on empowering the non-specialist physicians to reduce the time to intervention for patients with acute stroke in resource limited settings, making more patients eligible for time sensitive interventions. We recommend larger randomized controlled trials and qualitative research in LMICs to study the impact of similar devices in the clinical workflow on patient outcomes.

## Supporting information

S1 Appendix STARD guidelines. (DOCX)

S2 Appendix Regression analysis. (DOCX)
