# Supplementary material for: Artificial Intelligence-based automated CT brain interpretation to accelerate treatment for acute stroke in rural India: An interrupted time series study
Source: PLOS Glob Public Health. 2024 Jul 24;4(7):e0003351. doi: 10.1371/journal.pgph.0003351 (PMC11268585; doi:10.1371/journal.pgph.0003351)
Supplement: S2 Appendix — (DOCX) [file pgph.0003351.s002.docx]

# **Supporting information (S2)**

## **Regression analysis**

A multivariable regression model was fitted with TTI as the dependent variable and age in years, gender, intervention type (mannitol or blood-thinner) and phase of the study (pre- or post-AI phase) as the independent variables for investigating the association between intervention and median TTI using quantile regression method.

The median TTI was found to be significantly reduced in the post-AI period (regression coefficient [beta]: -21·79, standard error [SE]: 8·57, p = 0·01). Patients who received mannitol received it faster than patients who received any blood thinners (beta: -36·08, SE: 9·90, p < 0·001).

As secondary analyses, we also fitted the same dependent and independent variables and used quantile regression for 25^th^ and 75^th^ quantiles. The details of the regression analysis output are shown in the table below. The 25^th^ percentile TTI was also significantly different as per the regression analysis (beta: -26·43, SE: 7·81, p <0·001), but the 75^th^ percentile TTI although reduced in the post-AI phase 3, was not statistically significant (beta: -14·20, SE: 24·06, p = 0·56).

All three quantile regression models had 174 degrees of freedom with 169 residuals.

**S2 Table: Regression analysis output of quantile regression models.**

| **Variable** | **QR 50%** | | | **QR 25%** | | | **QR 75%** | | |
| --- | --- | --- | --- | --- | --- | --- | --- | --- | --- |
|  | **beta** | **SE** | **p** | **beta** | **SE** | **p** | **beta** | **SE** | **p** |
| Age | 0·792 | 0·416 | 0·03 | 0·357 | 0·348 | 0·31 | 1·200 | 0·784 | 0·13 |
| Gender (Male) | -12·125 | 9·717 | 0·21 | -10·000 | 8·236 | 0·23 | -24·200 | 28·962 | 0·41 |
| Intervention Type (Mannitol) | -36.083 | 9·898 | <0·001 | -18·571 | 8·304 | 0·03 | -86·600 | 25·656 | 0·001 |
| Phase (post-AI) | -21.791 | 8·568 | 0·01 | -26·429 | 7·815 | <0·001 | -14·200 | 24·061 | 0·56 |

***Variable****: Independent variable*
***QR 50%:*** *50^th^ quantile regression*
***QR 25%:*** *25^th^ quantile regression*
***QR 75%:*** *75^th^ quantile regression*
***beta:*** *regression coefficient estimate*
***SE:*** *standard error estimated by bootstrapping*
***p****: p-value*
